# Preparation and Characterization of Fenofibrate-Loaded PVP Electrospun Microfibrous Sheets

**DOI:** 10.3390/pharmaceutics12070612

**Published:** 2020-06-30

**Authors:** Emese Sipos, Tamás Csatári, Adrienn Kazsoki, Attila Gergely, Enikő Bitay, Zoltán-István Szabó, Romána Zelkó

**Affiliations:** 1 Department of Drugs Industry and Pharmaceutical Management, University of Medicine, Pharmacy, Sciences and Technology of Targu Mures, Gh. Marinescu 38, 540139 Targu Mures, Romania; emese.sipos@umfst.ro (E.S.); csataritamas@yahoo.com (T.C.); 2 University Pharmacy Department of Pharmacy Administration, Semmelweis University, H-1092 Hőgyes Endre utca 7-9, 1085 Budapest, Hungary; kazsoki.adrienn@pharma.semmelweis-univ.hu (A.K.); zelko.romana@pharma.semmelweis-univ.hu (R.Z.); 3 Department of Mechanical Engineering, Faculty of Technical and Human Sciences, Sapientia Hungarian University of Transylvania, Corunca, 1C, 540485 Targu Mures, Romania; agergely@ms.sapientia.ro (A.G.); ebitay@ms.sapientia.ro (E.B.)

**Keywords:** electrospinning, microfibers, Positron Annihilation Lifetime Spectroscopy (PALS), dissolution enhancement, amorphization

## Abstract

Fenofibrate-loaded electrospun microfibrous sheets were prepared in an attempt to enhance the dissolution of the poorly soluble antihyperlipidemic agent and to improve its bioavailability. Physicochemical changes that appeared during the electrospinning process were monitored using a wide array of solid-state characterization techniques, including attenuated total reflectance Fourier-transformed infrared spectroscopy and positron annihilation lifetime spectroscopy, while fiber morphology was monitored via scanning electron microscopy. Dissolution studies carried out both in 0.025 M sodium dodecyl sulfate and in water revealed an immediate release of the active agent, with an approximately 40-fold release rate enhancement in water when compared to the micronized active agent. The dramatic increase in dissolution was attributed partially to the amorphous form of the originally crystalline active agent and the rapid disintegration of the electrospun microfibrous sheet due to its high surface area and porosity. The obtained results could pave the way for a formulation of the frequently used antihyperlipidemic agent with increased bioavailability.

## 1. Introduction

As high-throughput methods emerge in the process of drug discovery, numerous promising candidates are appearing that present high affinity for the identified molecular targets. However, the chase for highly potent drugs often comes at the cost of undesirable physical properties, such as high lipophilicity, high molecular weight, and low solubility [1,2]. Drugs of low solubility account for almost 75% of all drug candidates, further increasing the burden on pharmaceutical technologists trying to deliver adequate enabling formulations for these compounds [3,4]. Over the years, various formulation strategies have been successfully applied for low-solubility drugs, such as particle-size reduction, lipid-based vehicles, preparation of solid dispersions, salt formation, amorphization, co-crystal formation, and cyclodextrin complexation [5,6,7].

**Figure 1:**
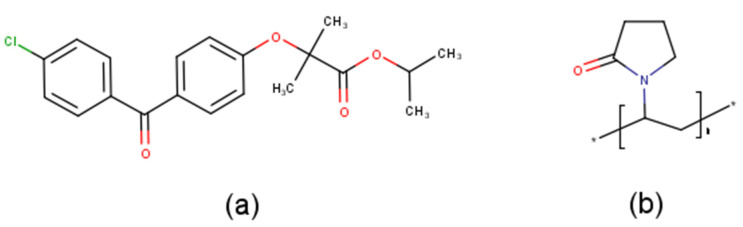
Chemical structures of (**a**) FEN and (**b**) PVP.

Fenofibrate (FEN, Figure 1a) as a model drug of extremely low solubility, is an ester derivative of the pharmacologically active fibric acid, which acts as a peroxisome proliferator-activated receptor-α (PPARα) agonist, reducing concentrations of plasma triglycerides and improving the ratio of high-density lipoprotein/low-density lipoprotein choresterol [8]. Unfortunately, among the numerous fibric acid derivatives available worldwide, FEN exhibits the lowest and most variable bioavailability. The molecule is neutral, highly lipophilic (logP = 5.4), and virtually insoluble in water [8], belonging to Biopharmaceutical Classification System (BCS) Class II. For BCS Class II drugs, solubility is the rate-limiting step in absorption. In order to overcome this problem, numerous fenofibrate formulations have been developed, and an excellent review [9] was also published about the available fenofibrate and fenofibric acid formulations Versatile types of formulations, e.g., micronized [10,11], microcoated [12,13], insoluble drug delivery microparticulate formulations [14], self-emulsifying drug delivery systems [15,16,17], nanoparticulate formulations [18,19,20,21], thin film freezing [22], incorporation in mesoporous silica [23,24,25,26,27,28], or carbon [29] have appeared in the last decade.

In recent years, electrostatic spinning (electrospinning) has emerged as the most dynamically growing technique for the generation of artificial fibrous architectures [30,31]. For drug delivery applications, fibers are produced usually from drug-loaded, viscous polymeric solution by applying high voltage, during which liquid droplets are electrified for the generation of initial jets, followed by stretching and elongation to produce fibers [30,32]. The obtained non-woven fibrous mats are characterized by large surface areas, porous structure, and the possibility of controlling the crystalline–amorphous transition of the active ingredient [32,33]. Owing to these advantages, nanofiber-based drug delivery systems are also under intensive study as a promising way to develop oral, fast-dissolving pharmaceutical dosage forms [32,34,35,36,37]. Besides the pharmaceutical and tissue engineering applications of nanofibrous systems, they are frequently applied for a wide variety of other purposes as well, e.g., filtration, affinity membranes and recovery of metal ions, catalyst and enzyme carriers, and energy storage [38,39].

The aim of this study was to prepare a fenofibrate-loaded drug delivery system based on polyvinylpyrrolidone (PVP, Figure 1b) fibers with the aid of electrospinning, intended for fast drug release in the buccal cavity. The microstructural characterization of prepared fibrous meshes, including the tracking of the physicochemical changes of electrospun fibers, was also in the focus of the present work.

## 2. Materials and Methods

### 2.1. Materials

The micronized FEN was a kind gift from a local pharmaceutical company in Tirgu Mures, Romania. PVP (Plasdone K-29/32) was obtained from ISP Technologies/Ashland, (Wayne, NJ, USA). Kolliphor RH 40, Kollisolv P124, Kolliphor EL were obtained from BASF (Limburgerhof, Germany); polysorbate 80 (Tween 80) was from Sigma-Aldrich (St. Louis, MO, USA); methanol, acetonitrile (gradient grade, Merck, Darmstadt, Germany), and sodium dodecyl sulfate (SDS, Merck, Darmstadt, Germany) were obtained through local vendors.

### 2.2. Preparation of Microfibrous Sheets by Electrospinning

First, 0.2 g FEN, 0.5 g polysorbate 80, and 2.4 g PVP were dissolved in 5 mL ethanol by magnetic stirring at 500 rpm using a JK SMS HS magnetic stirrer (JKI, Shanghai, China), until a clear viscous solution was obtained, which was transferred into a 20 mL plastic syringe. Drug-loaded microfibrous sheets were prepared by electrospinning using an in-house-assembled apparatus, employing the following parameters: 0.7 mL/h flow rate, provided by an Ascor AP 12 syringe pump (Ascor Med, Warsaw, Poland), the spinneret had a 1.5 mm internal diameter, a blunt metal needle that was connected to the 5 mL syringe with silicone tubing; the spinneret-to-collector distance was set to 7.5 cm and 15 kV voltage was applied. A 10–20 min continuous operation resulted in sheets of uniform diameter and thickness.

### 2.3. Morphology Investigation by Scanning Electron Microscopy (SEM)

The fiber mat morphology and the fiber diameter were investigated by SEM imaging, with the use of a JEOL JSM-5200 scanning electron microscope (JEOL, Tokyo, Japan) at 15 kV potential. The samples were used as is (without sputter coating) and were fixed by conductive carbon adhesive tape. SEM images were taken at different parts of the sample in order to determine the average fiber diameter. Diameters of 130 randomly selected individual fibers were measured using ImageJ software version 1.53C (US National Institutes of Health, Bethesda, MD, USA).

### 2.4. ATR-FTIR Spectroscopic Examinations

ATR-FTIR spectra were collected on Jasco FT/IR-4200 spectrophotometer (Jasco Inc., Easton, MD, USA) between 4000 and 400 cm^−1^ with an ATRPRO470-H single reflection accessory (Jasco) equipped with flat pressure tip. The spectral measurements were performed in absorbance mode. The 100 scans at a resolution of 4 cm^−1^ were co-added by the FTIR software (Spectra Manager-II, Jasco). FEN, PVP K-29/32 and polysorbate 80 were measured as single compounds and as physical mixtures; afterwards, spectra of the drug-loaded microfibers were also recorded.

### 2.5. Determination of Drug Content of FEN-Loaded Microfibers

Drug content of the microfibrous mats was determined using a Shimadzu UV-1601PC UV-VIS spectrophotometer (Shimadzu, Tokyo, Japan). Approximately 10 mg microfiber was accurately weighted in a 50 mL volumetric flask, dissolved in 25 mM sodium dodecyl sulfate solution, and completed to sign. Absorbances of the prepared solutions (*n* = 4) were measured at 289 nm in a 10 mm quartz cuvette (Merck, Germany), and concentrations were calculated on the basis of a calibration curve.

### 2.6. Dissolution Studies

Dissolution tests were carried out in an Erweka DT-80 dissolution apparatus (Erweka GmbH, Heusenstam, Germany) equipped with rotating baskets (Apparatus 1). The temperature of the dissolution medium was maintained at 37.0 ± 0.5 °C, and rotation speed was set at 100 rpm. Dissolution studies were performed in two separate dissolution media, both at a volume of 500 mL: water and 0.025 M sodium dodecyl sulfate. Sampling was performed manually at 1, 3, 5, 10, 20, and 30 min, by withdrawing 3 mL of samples with a syringe. The aliquots were filtered and the dissolved FEN content was determined spectrophotometrically, using a Shimadzu UV-1601PC UV-VIS spectrophotometer at 289 nm, in a 10 mm quartz cuvette.

### 2.7. Positron Lifetime Measurements

Supramolecular characterization of samples was carried out using positron annihilation lifetime spectroscopy (PALS). For ortho-positronium (o-Ps) lifetime determination, carrier-free ^22^NaCl positron source of activity of 10^5^–10^6^ Bq, sealed between two thin Kapton^®^ foils, was used with a fast–fast coincidence system based on BaF2 /XP2020Q detectors and Ortec^®^ electronics. The fibrous samples or the physical mixtures were placed at either side of the source (forming a sandwich structure), and were finally wrapped in aluminum foil. Three parallel spectra were measured at each composition to increase reliability. After summarizing the parallels, spectra were evaluated using the RESOLUTION computer code [40]; the indicated errors are the deviations of the lifetime parameters obtained. Two lifetime components were found in each sample, and the longest component was identified as the o-Ps lifetime, which is related to the annihilation of the o-Ps atoms.

## 3. Results and Discussion

**Figure 2:**
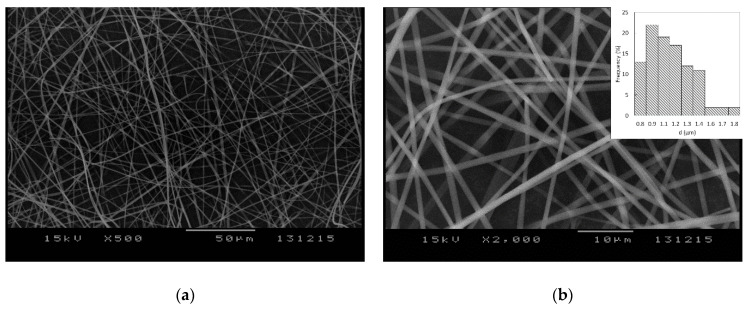
SEM images of the obtained electrospun microfibers at (**a**) 500× and (**b**) 2000× magnification and their diameter distributions (**b**).

Numerous preformulation experiments were performed in order to obtain a homogenous solution with adequate drug loading which would be suitable for electrospinning. PVP K-29/32 was selected as the fiber-forming polymer, which could be easily electrospun into microfibrous meshes when used in a concentration of 40% *w/v* in ethanol or methanol. However, FEN alone could not be dissolved in satisfactory concentration in the alcoholic PVP solutions; thus, several solubilizers (Kolliphor RH 40, Kollisolv P124, Kolliphor EL, and polysorbate 80) were tested in order to overcome this issue. Satisfactory results were obtained when FEN was dispersed in polysorbate 80 and dissolved afterwards in a 40% *w/v* PVP K-29/32 ethanolic viscous solution. The obtained solution was electrospun into a beadless, well-defined, randomly aligned fibrous structure, as observed in Figure 2. The obtained microfibers presented uniform structures with smooth surfaces. Mean fiber diameter as determined by SEM imaging was 1.10 ± 0.23 μm.

FEN content of the electrospun PVP-based microfibers was determined to be 6.65 ± 0.09% *w/w*, indicating homogenous FEN dispersion and repeatability among the different microfibrous mesh batches obtained through 10–20 min continuous electrospinning.

**Figure 3:**
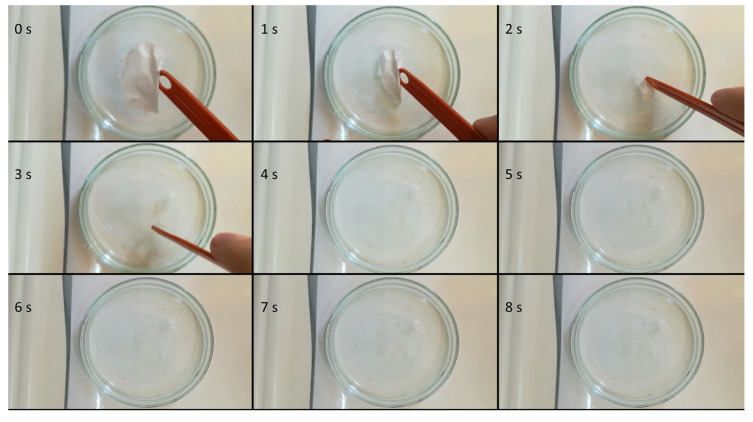
Sequential photographs of the disintegration process of the obtained microfibrous mesh.

Sequential photographs taken at an interval of 1 s depicting the immersion of an electrospun microfibrous mesh are shown in Figure 3. Due to its high surface area-to-volume ratio and high porosity, the fibrous material showed immediate disintegration when it came into contact with water. After disintegration, a clear solution was obtained, without the precipitation of FEN.

**Figure 4:**
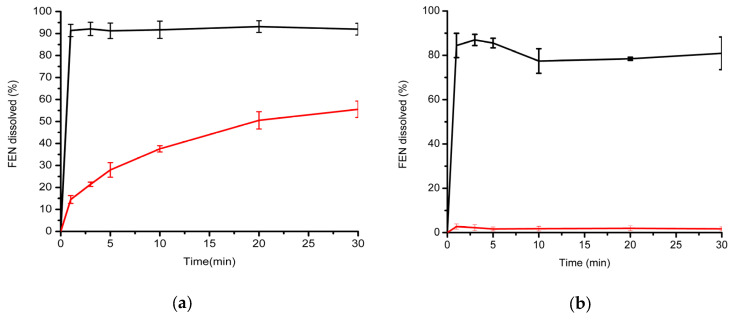
Comparative in vitro dissolution studies carried out in two different dissolution media: (**a**) 0.025 M aqueous SDS and (**b**) water. Black trace: electrospun FEN-loaded microfibers; red trace: micronized API.

Dissolution behavior of the prepared microfibers (test formulation) was compared directly to the micronized active ingredient (control) in two separate dissolution media. Since the model drug was neutral, water was selected as the first, more discriminative dissolution medium. Additionally, 0.025 M sodium dodecyl sulfate was selected as an alternative dissolution medium, as this is also the one suggested by the FDA dissolution database for micronized FEN-containing capsules. As shown in Figure 4, the microfibrous formulation outperformed the API in both cases: drug release was higher than 80% at the first sampling point for the microfibrous formulation, regardless of the dissolution medium employed. In the case of the micronized API, dissolution was extremely low, only approximately 2%, when using water as the dissolution medium. In comparison, dissolution from the microfibers remained at around 80% throughout the study, which translates to a very high, approximately 40-fold increase in dissolution rate of FEN when the active agent was incorporated into the microfibers.

Using the FDA-recommended 0.025 M SDS medium, dissolution of the micronized API was higher; however, the dissolution rate was slow: 14.53% at 1 min, which increased slowly to 55.54% at the end of the dissolution test, at 30 min. In comparison, FEN dissolution was immediate from the microfibrous sheets (91.39% at 1 min and 91.98% at 30 min). No precipitation of the active agent was observed after 30 min.

**Figure 5:**
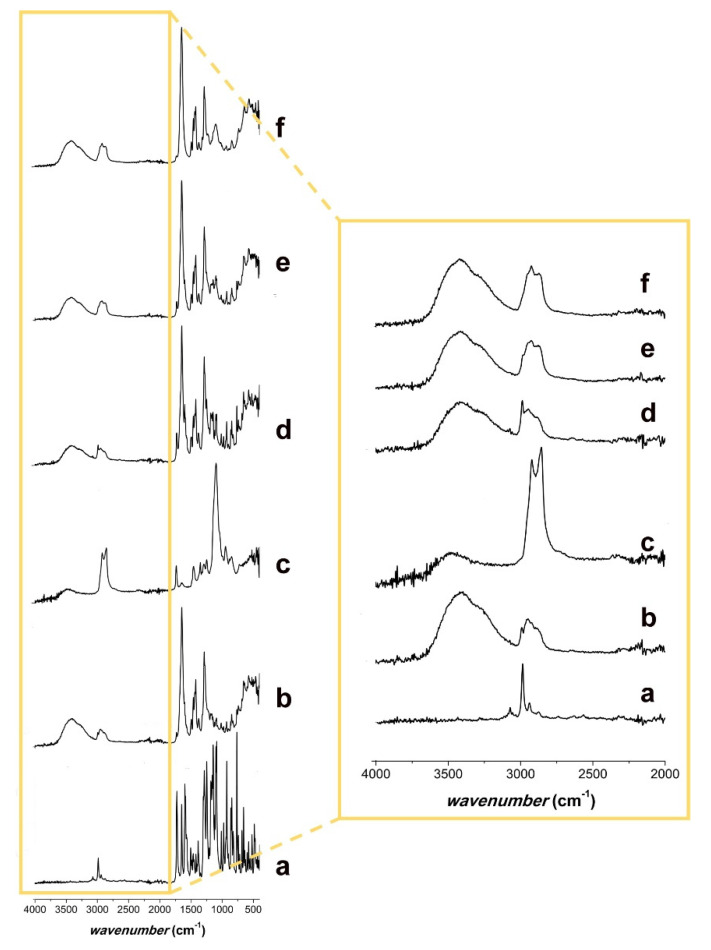
ATR-FTIR spectra of (**a**) FEN; (**b**) PVP K29/32; (**c**) polysorbate 80; (**d**) physical mixture of FEN and PVP K29/32; (**e**) physical mixture of FEN, PVP K29/32, and polysorbate 80; and (**f**) FEN-loaded microfibers.

The obtained ATR-FTIR spectra are shown in Figure 5. The spectrum of FEN (Figure 5a) showed peaks resulting from the CH of the aromatic rings at approximately 3050 cm^−1^, whereas the CH_3_ peaks presented at lower values of approximately 2950 cm^−1^. The characteristic peaks at 1730–1550 cm^−1^ corresponded to the carbonyl of the ketone and ester group, as shown in the chemical structure of the drug (Figure 1a).

The PVP K29/32 spectrum (Figure 5b) showed the CH_2_ peaks at 2900 and 1400 cm^−1^. At 3400 cm^−1^, the characteristic peak of the –OH group and its H-bonds was observed [41], which might result from a keto-amide equilibrium or the absorbed water of the powder. The peaks at 1650 cm^−1^ derived from the carbonyl of the pyrrolyl ring [42].

The spectrum of polysorbate 80 (Figure 5c) showed a characteristic peak for the –OH group at 3450 cm^−1^. At approximately 3000 cm^−1^ and 1400 cm^−1^, the peaks resulting from CH_2_ and CH_3_ were observed. The ester group showed a peak at approximately 1750 cm^−1^ and some more peaks around 1000–1300 cm^−1^, where the ether group was also found at 1200 cm^−1^.

The physical mixtures with and without polysorbate 80 (Figure 5d,e) presented all the characteristic peaks of the individual components.

Comparing the spectrum of the drug-loaded microfibers (Figure 5f) to that of the physical mixture with polysorbate 80 (Figure 5e), a missing signal in the large peak that goes from 2800 to 3000 cm^−1^ was noticed. Its wavenumber (2950 cm^−1^) was characteristic of the CH_3_ groups of FEN. Furthermore, the signal at 1600 cm^−1^ from the carbonyl stretching region, present in the FEN (Figure 5a) and both physical mixtures (Figure 5d,e), was missing from the drug-loaded microfibers’ spectrum. This might indicate that FEN was present in an amorphous form in the drug-loaded electrospun microfibrous samples [43,44,45].

PALS measurements were carried out with the aim of monitoring the supramolecular alterations through o-Ps lifetime changes of physical mixtures, and the electrospun neat and FEN-loaded fibrous samples.

**Figure 6:**
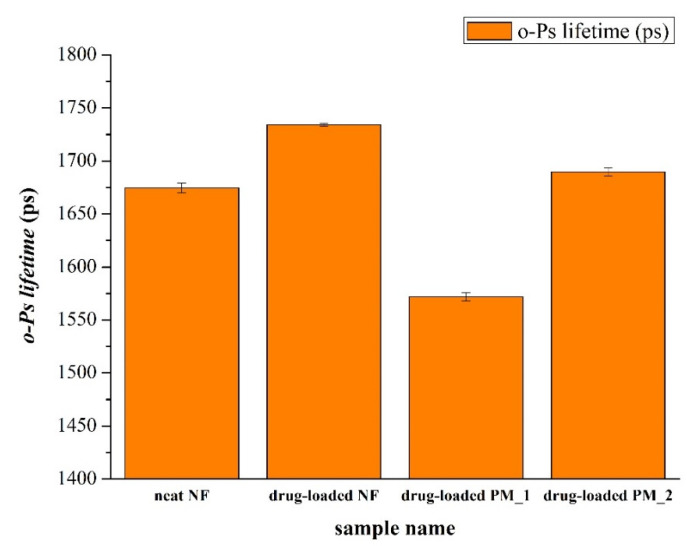
Average o-Ps lifetime values of various samples. neat NF: PVP-based microfibers without polysorbate 80 and FEN; drug-loaded NF: PVP-based microfibers with polysorbate 80 and FEN; drug-loaded PM_1: physical mixture containing PVP and FEN; drug-loaded PM_2: physical mixture containing PVP, fenofibrate, and polysorbate 80.

Along with the increase of the o-Ps lifetime values, the free volume holes within the polymeric chains also increased. Figure 6 illustrates that the samples with polysorbate 80 had remarkably higher o-Ps lifetime values than those of the other samples, either powder mixtures or fibers that lack polysorbate. This was due to the fact that the presence of polysorbate plasticized the fiber-base polymer both in the physical mixtures and in the fibrous samples, which could also have relevance for applications requiring the rigidity of the fibers to be decreased.

## 4. Conclusions

FEN-loaded, PVP-based microfibrous mats were prepared from polysorbate-80-containing viscous alcoholic solutions. The SEM studies of the prepared mats revealed homogenous, smooth filaments with a microfibrous structure. Complementary analytical methods provided intramolecular-level characterization (FTIR and PALS) of the electrospun fibrous samples. The mats presented rapid disintegration with immediate drug release, presenting an over 40-fold dissolution rate enhancement in water when compared to the micronized active ingredient. The obtained results could pave the way for further FEN formulations with enhanced biopharmaceutical characteristics.
